# Indifference to Professional Duty

**Published:** 1899-01

**Authors:** 


### Editorial.

### INDIFFERENCE TO PROFESSIONAL DUTY.

Loyalty to a calling, whether it be a trade or a profession, should be a part of the mentality of every individual, for without it both trade and professions are weakened. This is a truism, and needs no amplification.

How can professional duty or professional loyalty be defined? Not, certainly, by the ordinary commercial interpretation, for it assumes a higher phase in proportion to the exaltation of the calling. In all cases, however, whether this be high or low, it means that the individual having received a share of all the increment of knowledge previously acquired from those with whom he has been associated, should persistently defend that knowledge against all antagonizing forces, that it may be handed down to his successors in all its purity and without loss. This does not mean that he will selfishly grasp all the good there may be in it for his own advancement, but that that good may be so enlarged that those not alone of the household of faith may be benefited thereby, but, by reflection, the entire human family.

Indifference to professional duty is the weakness of dentists, and in proportion as their ethics fail to reach a high standard of rigid loyalty will it, to that extent, be ignoble.

It is well at the close of the year to take an account of the moral stock upon our professional shelves, and it is equally well at the opening of another twelve months to lay plans for future guidance. It is not always that these can be fulfilled, but the individual who fails to plan upon some system will never pursue a straight course, and can never reach any possible goal in life.

Has there been any gain upon the ethical side of our profession during the year just closed? It would be harsh to say there has not been any visible signs of a great moral upheaval; but it may be said, in truth, that if any change has taken place it has been towards a loss.

If loyalty to a calling makes that more worthy of respect, then it would seem that every individual enlisted under the dental banner would strive to make it worthy of the highest honor. Is this the case at present? It is feared the answer must be in the negative.

The writer was impressed with this fact in conversation recently with one of the bright young dentists of the period, and one who, should he live, will make a mark in his profession, that even he had not realized that there was something beyond scientific work due his calling, or that his indebtedness lay in the direction of a higher standard of professional ethics.

It was with him, as with the mass of the twenty thousand dentists of the United States, "Where shall I gain the most? If I write an article, where can I find a periodical having the largest circulation? Will this be found in 'The Universal Central Digester,' or in 'Capital and Interest?' If in one or both, then that is the place for my work to go." The question is rarely asked, "Where will I find the most intelligent circle of readers?" It is quantity always, and while quantity may give a semblance of truth to the idea that circulation insures a certain number of readers, it in reality means that so many journals decorate the office table, nothing more. The thinkers—and to be a thinker means a reader—are confined to a very narrow circle, and it is these that give character to any profession and make or unmake a young man in his struggle upward. The true goal for ambition is to seek this inner circle, and not that larger field of indifference that regards an advertisement as the most interesting portion of a journal.

From a lack of appreciation of the highest type of professional culture to that of indifference to duties connected with associative effort is but a step, the one being correlated with the other. It is assumed, and "Polk's Dental Register" enforces the assumption, that over twenty thousand dentists are operating to-day in the United States. According to this very reliable authority, there are in the United States and Canada one hundred and forty-six dental societies of all grades. Giving thirty members as an average to each, there must be four thousand three hundred and eighty active participants in society work, leaving fifteen thousand six hundred and twenty dentists who take no part whatever in elevating their calling. The number who are enrolled as members of societies by no means indicates the real working force. It is within the knowledge of every dental society man that not twenty per cent. attend the meetings, and probably not more than ten per cent. either write papers or speak in the discussions. It would be extremely difficult to determine how many of all the four thousand three hundred and eighty ever attempt original work.

All this is not stated in a pessimistic spirit, for it is recognized, as a necessary result, that all persons are not constituted alike; different tastes and different habits, combined with different degrees of intelligence, go to make up this chapter of indifference or interest in society work. While this is true, some effort should be made to instil a broader professional spirit; and as the profession has tried moral suasion without result, why may it not be well to adopt another plan, and make a constitutional provision requiring attention to society duties? With this exhibit it is not remarkable that but a small proportion of the twenty thousand are subscribers to the dental journals.

With those of real intelligence in the dental profession—those who control societies and have, in season and out of season, labored that dentistry might advance to a position worthy of its duty to humanity—there is to be found a class that seem unable to distinguish between the ethical side of society work and that of commercialism. This class will sell their proceedings to the highest bidder, and give the cold shoulder to the professional journal, simply because the latter, struggling for a higher moral standard, fails to have the millionaire's capital behind it. It is to the disgrace of dentistry that this exists. It has been barter and sale from the National Association down through the subordinate organizations, with the exception of the few associations that refuse to be tempted by "the thirty pieces of silver" to betray their profession. It is through the unselfish efforts of this small body that the dental profession has continued to live, and it is to be hoped that their labors will eventually neutralize and render ineffective the poison that is at present sapping its vitality.

We have a still higher work before us, and that is the establishment of an organization representing the best thought, the best work, and the highest grade of men in our calling. When this is accomplished, then will be raised a standard for all men to follow, and the present indifference will vanish under the power of a great moral force. This will come, it must come, and not until then will dentistry be worthy the name of a profession and be an honor to our civilization and an increased blessing to humanity.

